# Reprogramming of Tumor-Associated Macrophages with Anticancer Therapies: Radiotherapy versus Chemo- and Immunotherapies

**DOI:** 10.3389/fimmu.2017.00828

**Published:** 2017-07-14

**Authors:** Géraldine Genard, Stéphane Lucas, Carine Michiels

**Affiliations:** ^1^URBC – NARILIS, University of Namur, Namur, Belgium; ^2^Laboratory of Analysis by Nuclear Reaction (LARN/PMR) – NARILIS, University of Namur, Namur, Belgium

**Keywords:** tumor-associated macrophages, reprogramming, polarization immunotherapy, chemotherapy, radiotherapy, nuclear factor kappa B, reactive oxygen species

## Abstract

Tumor-associated macrophages (TAMs) play a central role in tumor progression, metastasis, and recurrence after treatment. Macrophage plasticity and diversity allow their classification along a M1–M2 polarization axis. Tumor-associated macrophages usually display a M2-like phenotype, associated with pro-tumoral features whereas M1 macrophages exert antitumor functions. Targeting the reprogramming of TAMs toward M1-like macrophages would thus be an efficient way to promote tumor regression. This can be achieved through therapies including chemotherapy, immunotherapy, and radiotherapy (RT). In this review, we first describe how chemo- and immunotherapies can target TAMs and, second, we detail how RT modifies macrophage phenotype and present the molecular pathways that may be involved. The identification of irradiation dose inducing macrophage reprogramming and of the underlying mechanisms could lead to the design of novel therapeutic strategies and improve synergy in combined treatments.

## Introduction

In 1863, Rudolf Virchow was the first to highlight the infiltration of leukocytes in tumor, thereby proposing a link between inflammation and tumorigenesis. Two centuries later, an inflammatory microenvironment within the tumor is part of the hallmarks of cancer (1). In fact, in emerging cancer disease, inflammation is a two-edge sword. On the one hand, the immune system can recognize tumor cells and kill them. On the other hand, chronic inflammation promotes cancer invasion, angiogenesis, and immunosuppression (2, 3). In this context, the immune system was assigned tumor immunoediting functions. The emergence of neoplastic cells induces an inflammatory environment, contributing to the rejection of the tumor (the *elimination phase*). However, the immune system establishes a selective pressure on cancer cells hence selecting resistant cells (the *equilibrium phase*). Finally, tumor evades from the immune system and moves into the *escape phase* (4). This last phase can occur through different mechanisms: reduced immune recognition, increased cancer cell resistance or survival, and immunosuppressive tumor microenvironment (5). In this process, macrophages act as an orchestrator of inflammation and are the main players of immunosurveillance. In the elimination phase, transformed cells are recognized and their antigens are presented to the effectors of the immune system by macrophages, promoting antitumor immunity (6). However, in the escape phase, macrophages play an important role in tumor progression by stimulating angiogenesis, metastasis, tumor growth, and immunosuppression notably through the secretion of polyamines, M-CSF, vascular endothelial growth factor (VEGF), IL-10, and transforming growth factor β (TGFβ) (5).

## Complicity of Tumor-Associated Macrophages (TAMs) in Tumor Progression

**Figure 1:**
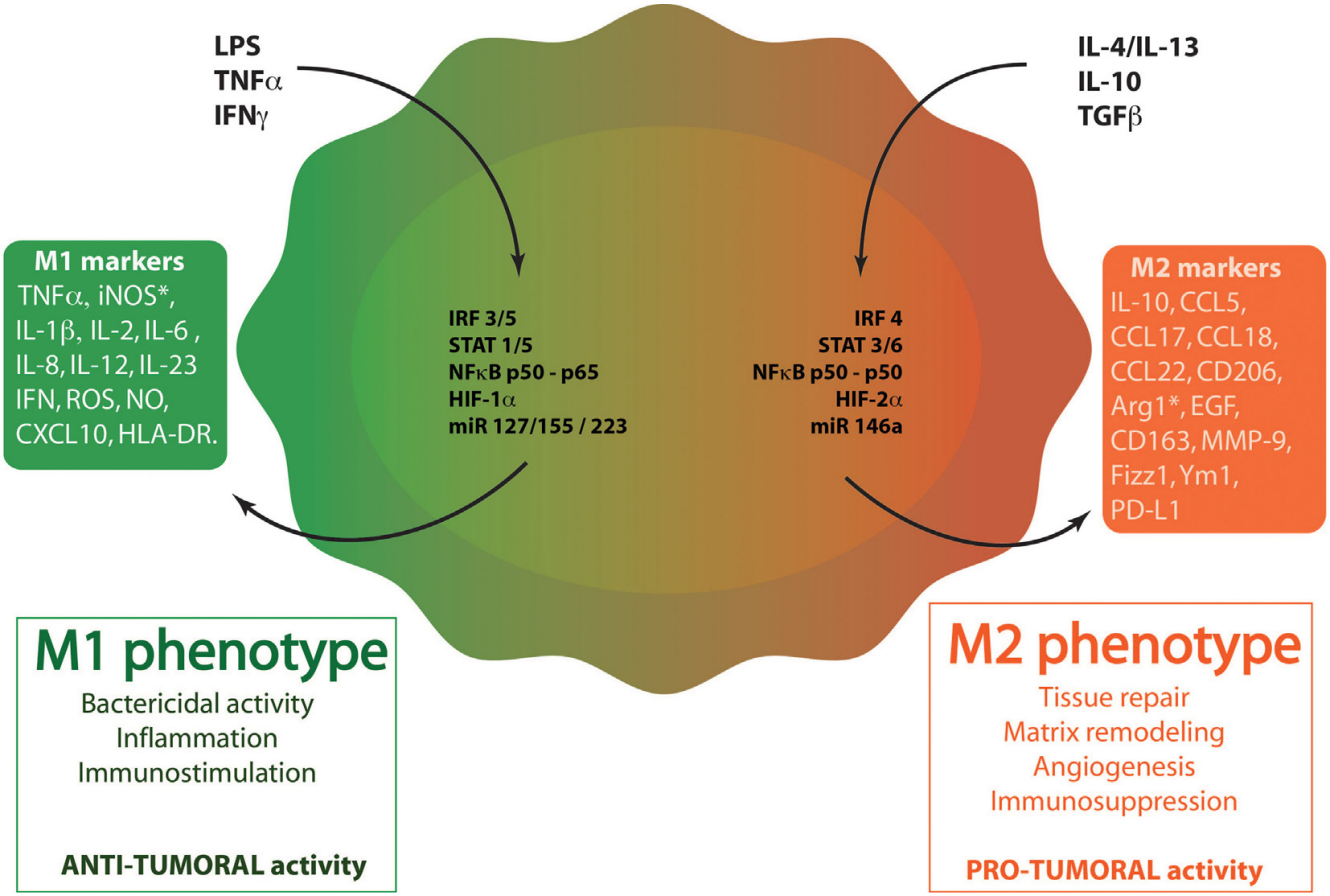
Macrophage polarization. Through the binding to their respective receptors, M1 stimuli [lipopolysaccharide (LPS), tumor necrosis factor α (TNFα), and interferon γ (IFNγ)] trigger the activation of several transcription factors. These factors include interferon-regulatory factor/signal transducer and activator of transcription (IRF/STAT) family members (IRF3, IRF5, STAT1, and STAT5), the active nuclear factor kappa B (NFκB) heterodimer (p50–p65) and HIF1. miR127, miR 155, and miR223 also regulates M1 polarization. When polarized in M1-like phenotype, macrophages produce specific cytokines (TNFα, IL-1β, IL-2, IL-6, IL-12, IL-23, IFNγ), chemokines (CXCL10) and other molecules [reactive oxygen species (ROS), nitric oxide (NO), inducible nitric oxide synthase (iNOS), human leukocyte antigen-cell surface receptor (HLA-DR)]. M1 phenotype plays key roles in inflammation, immunostimulation and an antibacterial and antitumoral responses. M2 stimuli [IL-4, IL-13, IL-10, and transforming growth factor β (TGFβ)] bind to ILR4α, ILR10, or TGFβR to induce M2-like phenotype in macrophages. These stimuli activate several transcription factors: IRF/STAT family members (IRF4, STAT 3, and STAT6), the inhibitory NFκB homodimer (p50–p50) and HIF2. miR14a also influences M2 polarization. When polarized in M2-like phenotype, macrophages produce specific cytokines (IL-10), chemokines (CCL5, CCL17, CCL18, CCL22), and other proteins (CD163, CD206, Arg1, MMP-9, Fizz-1, Ym-1, and PD-L1). M2 macrophages exert diverse functions, such as tissue repair, matrix remodeling, angiogenesis, immunosuppression, and favor tumor growth.

More than 50% of tumor-infiltrating cells are macrophages, named TAMs (7, 8). The recruitment and accumulation of TAMs into tumors are initiated by macrophage chemoattractants [e.g., CCL2/monocyte chemoattractant protein 1 (MCP-1), colony-stimulating factor 1 (CSF-1)] and it is well established that TAMs drive tumor progression (9, 10). In fact, cancer prognosis is closely linked to the number of TAMs with an inverse correlation: increased number of TAMs is associated with a reduced cancer patient survival (11). In healthy tissues, macrophages offer a remarkable plasticity to efficiently respond to environmental cues (12). In tumors, an identical plasticity is described: TAMs are educated by the tumor microenvironment, providing multiple phenotypes with a range of functions (13). TAM phenotypes can be featured as a linear scale where M1 and M2 phenotypes represent the two extremes, similarly to the T_H_1–T_H_2 classification (Figure 1). M1 macrophages are recognized as classically activated macrophages and show enhanced ability to phagocyte pathogens. More importantly, these cells have antitumoral properties. Macrophages can also be polarized into the M2 phenotype, the alternative activated state of macrophages. M2 macrophages are requested in infection-free healing circumstances and have pro-tumoral functions. The polarization of macrophages can be driven by different microenvironmental molecules and leads to the production by the macrophages of different cytokines and chemokines. M1 macrophages are activated during acute inflammation by toll-like receptor (TLR) ligands [lipopolysaccharide (LPS)] or T_H_1 cytokines [interferon γ (IFNγ)–tumor necrosis factor α (TNFα)]. M1 macrophages display an enhanced production of pro-inflammatory cytokines (TNFα and interleukins: IL-1β, IL-2, IL-6, IL-12, IL-23), reactive oxygen species (ROS), nitric oxide (NO) and present antigens *via* major histocompatibility complex class II molecules (14). Stimulation of macrophages with IL-4/IL-13, IL-10, TGFβ, or glucocorticoids leads to the M2 phenotype and the subsequent production of anti-inflammatory cytokines (IL-10, TGFβ) that have an inhibitory effect on cytotoxic CD8^+^ T cells. Macrophages with M2 phenotype also express cell surface scavenger receptor (CD206), hemoglobin receptor (CD163) and produce extracellular matrix (ECM) components (14–16). M2 macrophages facilitate the resolution of inflammation and promote tissue repair by T_H_2 response, tissue remodeling, and immune tolerance. They also favor tumor growth (17, 18). This phenotype can be subclassified into M2a, M2b, or M2c according to the roles these macrophages exert (19). In cancer disease, TAMs usually exhibit a M2 phenotype and participate to tumor angiogenesis, tumor invasion and metastasis, immunosuppression and cell activation. All these features led Qian and Pollard to classify TAMs in six functional subtypes: angiogenic, immunosuppressive, invasive, metastasis associated, perivascular, and activated macrophages (20).

## Molecular Pathways for M1–M2 Polarization

The polarization of macrophages is influenced by several mechanisms driven by transcription factors and miRNAs (Figure 1). The activation of macrophages into M1 or M2 phenotype is mainly induced by interferon-regulatory factor/signal transducer and activator of transcription (IRF/STAT) signaling pathways. IRF3, IRF5, STAT1, and STAT5 are responsible for driving M1 polarization while IRF4, STAT3, and STAT6 provide M2 activation signals (14, 21). Besides IRF/STAT transcription factors, hypoxia also influences macrophage polarization. The lack of oxygen can activate hypoxia-inducible factors (HIF) differently in M1 versus M2 macrophages. Indeed, T_H_1 cytokines are able to induce HIF1α stabilization, triggering M1 response whereas HIF2α is activated by T_H_2 cytokines in M2 macrophages. These differences rely on the ability of HIF1 and HIF2 to, respectively, activate or suppress NO synthesis (22). Furthermore, nuclear factor kappa B (NFκB) fulfills a central role in the pro-inflammatory macrophages (M1). Indeed, the active heterodimer NFκB (p50–p65) promotes the transcription of inflammatory genes while the inhibitory heterodimer NFκB (p50–p50) prevents the transcription of these genes in anti-inflammatory macrophages (M2). Other transcription factors including AP-1, Kruppel-like factor 4 and PPARγ can modulate the activated state of macrophages. Finally, miRNA also interfere with the polarization of macrophages. Among miRNAs of interest, miR127, miR155, and miR223 are key regulators of M1 polarization (23, 24). In contrast, miR146-a promotes M2 polarization while decreasing the expression of M1 markers (25).

## Targeting TAMs with Chemotherapy or Immunotherapy

**Figure 2:**
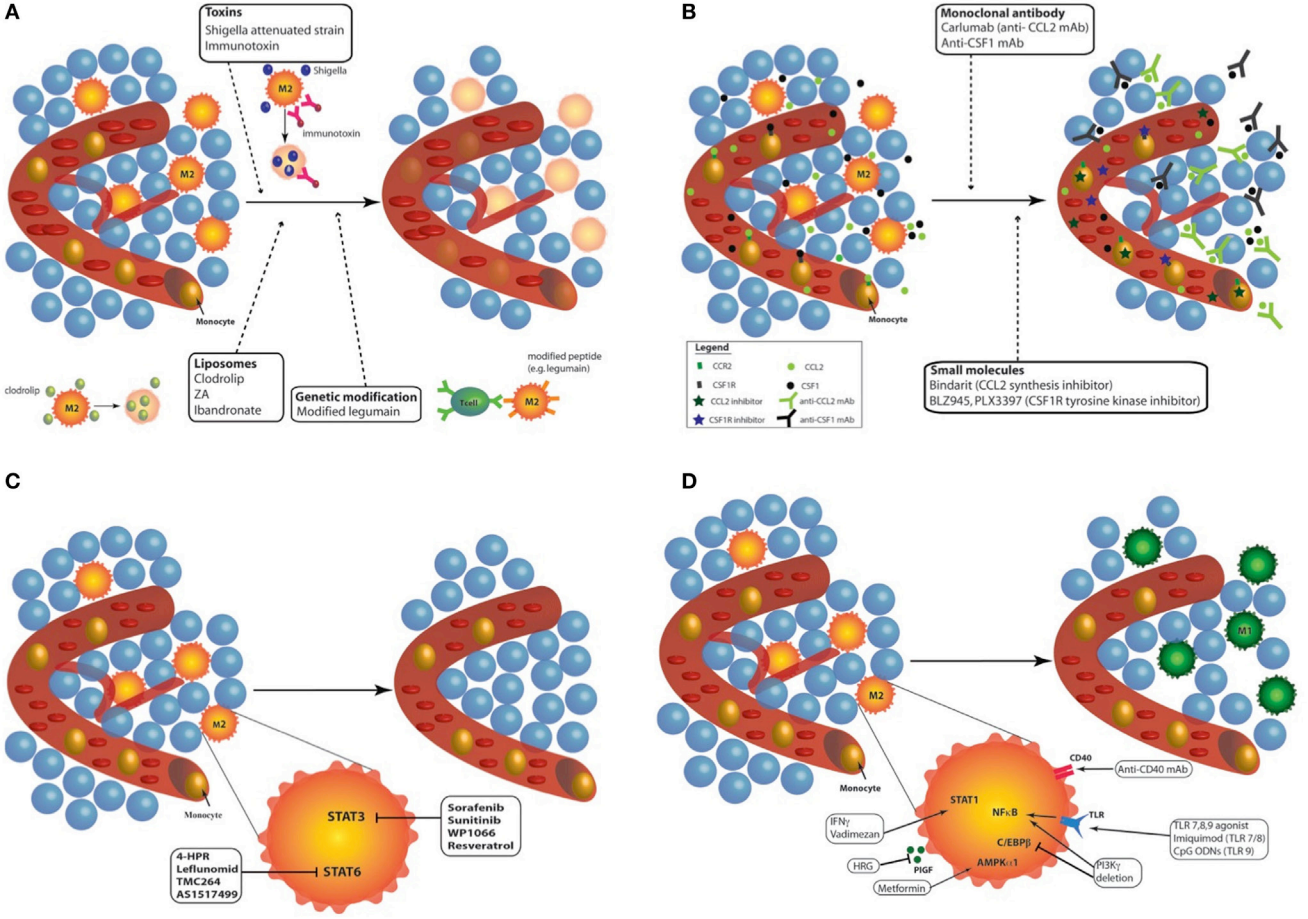
Targeting tumor-associated macrophages (TAMs) with chemo- and immunotherapies. Different approaches have been proposed to modulate TAMs: **(A)** *Depletion of TAMs*: different kinds of treatments are available to destroy TAMs in tumor: toxins (*Shigella flexneri* attenuated strain or immunotoxin), liposome containing bisphosphonates [clodrolip, zoledronic acid (ZA) or ibandronate], and peptide modification to induce cytotoxic lymphocyte activation (e.g., legumain). Depletion of macrophages in tumor induced effective tumor regression in mouse and patients. **(B)** *Inhibition of circulating monocyte recruitment into the tumor*: two main recruitment effectors can be targeted to inhibit the recruitment of monocyte to the tumor site: CCL2/C–C chemokine receptor type 2 (CCR2) and colony-stimulating factor 1 (CSF-1)/CSF1-R. The use of monoclonal antibody against CCL2 (e.g., Carlumab) or CSF-1 inhibits tumor growth in mouse models and humans. Another way to prevent monocyte recruitment to the tumor site is the use of molecule targeting CCL2/CCR2 (e.g., bindarit) or CSF1/CSF1-R (e.g., BLZ945, PLX3397) pathways. **(C)** *Blockade of M2 phenotype*: the blockade of M2 phenotype can be achieved by targeting two main transcription factors: STAT3 (sorafenib, sunitinib, WP1066, and resveratrol) and STAT6 (4-HPR, leflunomid, TMX264, and AS1217499). All these inhibitors provide tumor regression and inhibited angiogenesis. **(D)** *Enhanced activation of M1 macrophages or reprogramming of TAMs toward M1-like macrophages*: TAM reprogramming into M1 macrophages can be achieved through the stimulation of STAT1 (IFNγ, vadimezan), AMPKα1 (metformin), or nuclear factor kappa B [toll-like receptor agonists such as imiquimod or CpG-ODNs; phosphoinositide 3-kinase (PI3Kγ) deletion]. The inhibition of placental growth factor (PlGF) (HRG) and C/EBPβ (PI3Kγ deletion) also leads to effective reprogramming of TAMs toward M1-like macrophages. Finally, by stimulating CD40, monoclonal antibodies (mAbs) against CD40 similarly reprogram TAMs from M2 phenotype to M1 macrophages.

In response to microbial signals, tissue damage, cytokines and metabolic products, monocytes, and macrophages from healthy tissues are able to undergo reprogramming (12, 21, 26, 27). The pro-tumoral functions of TAMs and their ability to be reprogrammed, from M2-like macrophages toward M1 phenotype, make them an attractive target for anticancer therapies. Currently, different approaches have been proposed to modulate TAMs: (1) depletion of TAMs; (2) inhibition of circulating monocyte recruitment into the tumor; (3) blockade of M2 phenotype; and (4) enhanced activation of M1 macrophages or reprogramming of TAMs toward M1-like macrophages (27, 28). Each of these approaches will be detailed here under (Figure 2).

### Depletion of TAMs

Three alternatives are available to destroy TAMs in tumor (Figure 2A). The first way is the use of chemical compounds, especially bisphosphonates, such as clodronate encapsulated in liposomes [clodrolip (CL)]. These liposomes containing clodronate are phagocyted by macrophages and are disrupted by lysosomal processes, leading to the release of clodronate in the cells. Clodronate is metabolized to an analog of ATP, cytotoxic for macrophages (29). CL is currently used in immune research and clinical trials to eliminate macrophages and phagocytes in multiple malignancies. For example, TAM depletion with CL impaired tumor engraftment, reduced tumor growth, and favored mouse survival in a chronic lymphocytic leukemia model (30). However, despite its effective tumor regression effects, it also induced severe side effects such as sensitivity toward infections and weight loss in mice and patients (31–34). A similar approach was developed using zoledronic acid (ZA), which inhibited tumor progression, angiogenesis, and metastasis in association with sorafenib in two human hepatocellular carcinoma mouse models (34). ZA depleted matrix metalloproteinase 9 (MMP-9) expressing TAMs (M2-like macrophages) but also impaired the differentiation of myeloid cells into TAMs (35). However, ZA, as ibandronate (another bisphosphonate), has generated conflicting results.

The targeting of MMP-9 positive macrophages was also obtained with dasatinib, a tyrosine kinase inhibitor well tolerated by patients and approved by food and drug administration (FDA) for chronic myeloid leukemia (36). However, dasatinib has been reported to inhibit other immune cells as natural killer (NK) cells and T cells, then aborting tumor immune response. Finally, trabectedin (ET-743), another drug approved by FDA, exhibited a cytotoxic effect against TAMs and showed antitumor activity. This drug was shown to not damage lymphocyte subgroups. This is due to the differential expression of TNFα-related apoptosis-inducing ligand receptor (TRAIL) (TNFα-related apoptosis-inducing ligand) receptor by the different types of leukocytes: trabectedin targets TAMs while human NK cells and CD8^+^ lymphocytes were resistant to TRAIL-mediated toxicity. In fact, blood monocytes and TAMs expressed higher level of TRAIL-R2 compared to NK cells and CD8^+^ lymphocytes (37, 38).

A second alternative is the use of toxin-conjugated monoclonal antibodies (mAbs) or attenuated bacteria that kill macrophages. An anti-scavenger receptor A (CD204) antibody conjugated to the saporin toxin reduced the number of vascular leukocytes and inhibited tumor progression in a murine ovarian cancer model (39). An immunotoxin composed of portions of anti-folate receptor beta antibody conjugated to *Pseudomonas* exotoxin A was also used to deplete TAMs: reduced tumor growth was observed in nude mice bearing C6 glioma xenografts (40). In the same line, *Shigella flexneri* is a bacterial pathogen inducing the apoptosis of macrophages. Galmbacher and his colleagues developed an attenuated strain of *S. flexneri* to infect breast cancer-bearing mice. The treatment led to TAM depletion and to a complete tumor regression in tumor-bearing mice (41).

The last alternative is the activation of cytotoxic T lymphocytes against macrophages. Legumain is an asparaginyl endopeptidase that contributes to the degradation of the ECM and to angiogenesis. TAMs have been found to express abundant amounts of legumain (42). This discovery led Smahel and his team to modify the gene sequence of legumain to enhance the efficacy of immunization against legumain. These modifications induced reduced legumain maturation and impaired cellular localization, and resulted in T helper (CD4^+^) and cytotoxic (CD8^+^) lymphocyte-dependent elimination of TAMs (43).

Depletion of macrophages has thus met successful results in the tumor regression in mouse models and patients. However, a systemic depletion of macrophages obviously renders the organism more sensitive to infections and other aggressions. Therefore, these types of treatments need to be localized to the tumor site.

### Inhibition of Circulating Monocyte Recruitment into Tumor

The inhibition of CCL2/C–C chemokine receptor type 2 or CSF1/CSF1-R pathways by several methods was shown to efficiently induce tumor regression (Figure 2B). CCL2 is a chemokine produced by cancer cells that is responsible for the recruitment of monocytes at the tumor site. CCL2 overexpression in tumor is correlated with macrophage infiltration and poor prognosis in human cancers (44–46). Furthermore, CCL2 is a key actor of metastasis since it enhances the retention of metastasis-associated macrophages in breast cancer metastasis. CCL2 blockade was reported to block the mobilization of monocytes from the bone marrow to the blood in a murine breast cancer model (47). The inhibition of CCL2 by an anti-CCL2 monoclonal antibody (e.g., carlumab) or through the inhibition of its synthesis (e.g., bindarit, trabectedin) prevented the recruitment of macrophages into the tumor site. Most of these treatments are now tested in clinical trials. Carlumab, a human monoclonal antibody against CCL2, was safely used in metastatic castration-resistant prostate cancer (48) and in a phase Ib study in association with other chemotherapy agents (docetaxel, gemcitabine, paclitaxel or carboplatin and pegylated liposomal doxorubicin) (49). This monoclonal antibody led to an efficient depletion of macrophages into the tumor and can significantly delay tumor regrowth following chemotherapy (49). Besides mAb targeting CCL2, other compounds (e.g., bindarit, trabectedin) were found to inhibit the synthesis of CCL2/MCP-1. Bindarit reduced TAM and myeloid-derived suppressor cell infiltration in a breast cancer model and resulted in impaired metastatic disease in a prostate cancer model (50). This treatment also targeted angiogenesis and tumor growth in human melanoma xenografts (51). In addition to deplete TAMs, trabectedin is used to inhibit monocyte recruitment. This drug targets CCL2 synthesis by interacting with the transcription machinery (52) but also modulates the DNA repair machinery (53). In clinic, it is used to treat ovarian and breast cancer as well as soft tissue sarcomas (53, 54).

Colony-stimulating factor 1/CSF1-R signaling drives the recruitment and the differentiation of TAMs toward a M2 phenotype in tumor. CSF1-deficient mice showed a 50% decrease in macrophage infiltration while neutrophil infiltration was increased during tumor progression in a mouse model of pancreatic islet cancer (55). mAbs and small molecules targeting CSF1 (mAb anti-CSF1) or CSF1-R (BLZ945, emactuzumab, PLX3397) were subjected to numerous studies and were shown to deplete macrophages in a tissue-specific manner (56, 57). Overall, CSF1-R inhibitors deplete TAMs and abolish tumor growth, angiogenesis, and metastasis. BLZ945, a highly selective small molecule inhibitor of CSF1-R tyrosine kinase, attenuated the turnover rate of TAMs while increasing the number of CD8^+^ T cells in murine cervical and breast carcinoma models (56). In addition, blockade of CSF-1/CSF1-R by mAb anti-CSF1 or with PLX2297 (a small molecule targeting the tyrosine kinase domain of CSF1-R) was reported to reprogram remaining TAMs at the tumor site to support antitumor immunity in pancreatic cancer mouse models (58). PLX3397 also delayed the recurrence of glioblastoma after radiation by modifying the recruitment and polarization of myeloid cells in an intracranial xenograft model (59). However, orally administered PLX3397 showed no efficacy for human glioblastoma in a phase II study (60). Other chemoattractants for macrophages such as VEGF, C–X–C motif chemokine 12, CCL4 are also under investigation and may be considered as potential targets to inhibit macrophage recruitment and hence tumor progression.

### Blockade of M2 Phenotype

Two main transcription factors have been largely reported to block M2 polarization: STAT 3 and STAT6 (Figure 2C). Tyrosine kinase inhibitors (sunitinib and sorafenib) inhibit STAT3 in macrophages. Sorafenib was shown to restore the secretion of IL-12 while suppressing IL-10 expression in prostaglandin E2-conditioned macrophages, indicating a reverse effect on the immunosuppressive cytokine profile in TAMs (61). Inhibition of STAT3 with WP1066 reversed immune tolerance in patients with glioblastoma multiforme. This treatment stimulated the secretion of pro-inflammatory cytokines and activated T cells (62). In the same line, resveratrol has also been used to suppress M2-like polarization of TAMs with parallel inhibition of tumor growth in a mouse lung cancer xenograft model. The blockade of M2-like polarization of TAMs by resveratrol was linked to the decreased activity of STAT3 (63).

Similarly, fenretinide (4-HPR) inhibited the phosphorylation of STAT6 and skew M2 polarization. In a colorectal mouse model, the effects were accompanied by a reduction in the number of M2-like macrophages in tumor and by an inhibition of angiogenesis (64). Other inhibitors of STAT6 activation (TMC264, AS1517499) were developed but no one underwent clinical studies (65–67).

### Enhanced Activation of M1 Phenotype or Reprogramming of TAMs toward M1-Like Macrophages

Several options are currently used to select M1 phenotype from TAMs or to reprogram TAMs from M2 to M1 phenotype: TLR agonists, mAb targeting inhibitory proteins of M1 phenotype as well as other compounds (Figure 2D). TLR agonists represent a promising antitumor therapy. However, different agonists have been shown to promote different immune responses. Imiquimod is a ligand of TLR7 and acts mainly by increasing the number of infiltrating CD43^+^ lymphocytes. This TLR agonist was found to induce the nuclear translocation of NFκB in J774A macrophages, leading to the production of pro-inflammatory proteins, such as TNFα, IL-6, IL-12, and CCL2 (68). The combination of topical imiquimod with low doses of cyclophosphamid and radiotherapy (RT) revealed a synergic antitumor effect and an increased survival response. This allowed the prevention of recurrence, with tumor rejection over 2 months after the end of treatment in a cutaneous breast cancer model (69). Multiple phase I and phase II clinical trials for imiquimod resulted in the use of topical application of imiquimod in clinic for skin metastasis and carcinomas. Synthetic unmethylated cytosine-guanine (CpG) oligodeoxinucleotides (CpG-ODNs) also offered high immunostimulatory activity. These molecules act by enhancing the production of pro-inflammatory cytokines, such as TNFα, IL-6 and IL-12, in macrophages and by upregulating NFκB activity in these cells. Indeed, these nucleotides are frequently found in viral and bacterial genomes and are recognized as pathogen-associated molecular patterns by TLR9 (70–72). Multiple studies aimed to improve the delivery of these CpG-ODNs by coupling them to gold nanoparticles for example (73). Combination of CpG-ODNs with other therapies, such as anti-CD40 exhibited promising results associated with the repolarization of TAMs (74, 75), even in poorly immunogenic cancer models such as a preclinical glioma model (76).

Another alternative to favor cytotoxic functions of TAMs is the stimulation of CD40 with mAbs. CD40 mAbs have demonstrated antitumor T-cell responses in mouse models of cancer and in clinical trials (77). Macrophages also express CD40 in their plasma membrane. Anti-CD40 mAbs were shown to promote macrophage tumoricidal activity, especially through enhanced secretion of NO and TNFα. Hence, it could induce CD8^+^ T cell-dependent inhibition of tumor growth and metastasis (78–80). Clinical studies revealed objective tumor response in solid tumors (81). Since anti-CD40 mAbs can induce programmed death ligand 1 (PD-L1) upregulation in TAM and tumor-infiltrating monocyte plasma membrane, the blockade of PD-L1 axis combined with anti-CD40 and anti-CTLA-4 (anti-cytotoxic T-Lymphocyte-associated protein 4) mAbs showed extensive survival in colon and breast cancer models (77). Finally, MARCO is a pattern recognition scavenger receptor and is expressed by immunosuppressive TAMs. The targeting of this receptor is a new promising way to treat mammary carcinoma, colon carcinoma, and melanoma through the reprogramming of immunosuppressive TAMs toward a pro-inflammatory phenotype and by increasing tumor immune response (82).

A third alternative to reprogram TAMs is the use of different chemical compounds. The most famous one is IFNγ (83), approved by the FDA. In the same line, a small flavonoid-like compound, vadimezan (DMXAA), was found to repolarize macrophages in M1 phenotype. Reprogrammed macrophages then released cytokines and chemokines, including high local levels of TNFα, to induce a subsequent CD8^+^ T cell activation (84). Vadimezan has been the subject of numerous preclinical studies and clinical trials. Alternatively, macrophage phosphoinositide 3-kinase γ (PI3Kγ) was shown to control a critical switch between immune suppression and activation. Selective deletion of PI3Kγ simultaneously activates NFκB and inhibits C/EBPβ in macrophages. In combination with anti-PD-L1, PI3Kγ depletion promoted tumor regression and prolonged survival in head and neck, lung, and breast cancer murine models (85). The plasma protein histidine-rich glycoprotein (HRG) is also able to block TAMs into a M1 phenotype through the downregulation of the expression of the placental growth factor (PlGF), a member of the VEGF family. In mice, HRG promoted antitumor immune responses and normalization of the vessel network (86). Finally, metformin was shown to skew TAMs polarization into a pro-inflammatory phenotype, partially through AMPKα1 activation. This effect was concomitant with a decrease in the number of metastases in Lewis lung cancer intravenous model (87).

While depletion of macrophages induced toxicities in mouse models and humans, the inhibition of macrophage/monocyte recruitment is one of the most used therapies in clinical studies. This type of therapy is usually combined to chemotherapy or RT and generated encouraging results in patients. On the other hand, the reprogramming of TAMs by chemotherapies or immunotherapies seems another very attractive way to target macrophages in tumors. However, there is a huge need for clinical studies to confirm these preclinical data in humans. Furthermore, it has to be noted that these treatments need to be localized to avoid an activation of M1 macrophages outside of the tumor and a systemic inflammatory response.

## Targeting TAMs with RT

In addition to target TAMs using chemo- and immunotherapies, it is also possible to influence the macrophage polarization with RT. Herein, we explore how low linear energy transfer (LET) radiotherapies (X-rays and γ-rays) can repolarize TAMs *in vitro* and *in vivo*. In a first part, the doses which have to be applied to the tumor in order to induce TAM reprogramming will be described and in a second part, the mechanisms involved in RT-induced reprogramming will be detailed.

### Macrophage Radioresistance and Recruitment after RT

Macrophages are one of the most radioresistant cells in humans (7). This radioresistance is brought by a huge production of anti-oxidative molecules, such as manganese superoxide dismutase (MnSOD), a scavenger of superoxide (${\text{O}}_{2}^{-}$) ions. The high expression of MnSOD confers cellular resistance against damaging effects. These damaging effects are mainly produced by radiation-induced radicals, such as ROS or reactive nitrogen species (RNS). Indeed, the expression of MnSOD was increased after irradiation in THP-1, HL60, and KG-1 myelocytic cell lines. The mechanism of radioresistance conferred to macrophages was shown to be dependent on TNFα signaling. Indeed, endogenous production of TNFα is required for MnSOD expression following ionizing radiation (IR) (88–90). This TNFα-induced MnSOD expression is mediated by PKC-dependent activation of cAMP-responsive element-binding protein-1/ATF-1-like factors and would be dependent on NFκB activation (90–92). Currently, the radioresistance provided by endogenous MnSOD to macrophages is mimicked in healthy cells using MnSOD plasmid/liposome gene therapy to confer radioresistance to healthy cells (93–95).

Radiotherapy is used for more than 50% of cancer patients and showed tumor regression in most of the cases (33, 96). However, in addition to the intrinsic radioresistance of macrophages, IR also elicits a high recruitment of myeloid cells at the tumor site, possibly leading to tumor recurrence and tumor regrowth (59). Depletion of macrophages by liposomal clodronate before IR promoted the antitumor effects of RT and highlighted the role of recruited TAMs in tumor regrowth (90). Macrophage recruitment after IR is mediated by CCL2 and CSF1. Indeed, RT stimulated CSF1 production in prostate cancer and was shown to be responsible for TAM accumulation (97). Similarly, the inhibition of CSF1 receptor with PLX3397 prevented the myeloid cells recruitment after IR, increased survival and postponed the recurrence of glioblastoma in intracranial xenograft models (59). RT also promotes macrophage infiltration, from the peritumoral environment to the tumor site, in a CCL2 dependent way (98, 99). However, to the best of our knowledge, there is no study combining RT to CCL2 inhibition described yet.

### Dose-Dependent Effects of Irradiation on Macrophage Reprogramming

Although IR slightly affects the viability of macrophages, radiotherapy modifies the macrophage phenotype. To analyze the effects of radiotherapy on macrophage reprogramming, we classified these effects according to the dose. According to the UNSCEAR (United Nations Scientific Committee on the Effects of Atomic Radiation), low dose of X-ray radiation are doses under 0.1 Gy, but clinically applied low doses are doses under 1 Gy (100). In this review, the irradiation doses were classified as followed: low doses as doses lower than 1 Gy, moderate doses (MDI) as doses ranging from 1 to 10 Gy, and high doses as doses higher than 10 Gy.

#### High Doses of Irradiation (HDI)

*In vitro*, M1 Raw264.7 macrophages were reprogrammed toward M2-like macrophages after HDI (20 Gy). Sustained M2 phenotype after irradiation resulted from NFκB p50 activation, leading to high IL-10 production and reduced TNFα secretion. These results were confirmed *in vivo* since HDI (3 Gy × 20 Gy over 3 days) promoted M2-like macrophages at the tumor site, in mice with Panc02 cell xenografts (101). In another study, high doses of X-rays (25 Gy in one shot or 60 Gy fractioned over 3 weeks) increased the number of M2-like TAMs in a murine model of prostate cancer (TRAMP-C1 cell line). This was evidenced by a low inducible nitric oxide synthase (iNOS) level in macrophages and by an increase in Arg1 and COX-2 mRNA expression. Such high doses induced angiogenesis and tumor growth in this cancer model (7). An increased number of M2-like macrophages after high doses of radiation (12 Gy) was also observed in an oral cancer model. The recolonization of tumor by M2-like macrophages elicited the secretion of pro-angiogenic factors that contribute to neoangiogenesis, favoring tumor growth (102). In addition, Seifert et al. similarly described accelerated tumor growth after irradiation (12 Gy) of murine pancreatic tumor models. This was driven by an early infiltration of M2-polarized TAMs into the tumor after radiotherapy and resulted in a subsequent T-cell suppressive response. This T cell response was linked to the upregulation of PD-L1 expression in tumor. The authors showed that RT accelerated the progression of pancreatic dysplasia to invasive carcinoma but also promoted tumor growth in invasive pancreatic ductal adenocarcinoma (PDA). Indeed, a decrease in iNOS, IRF5 and H2eb1 mRNA expression and a higher expression of Arg1, CD206, and PD-L1 were observed in TAMs from pancreatic dysplasia and PDA irradiated with hypofractionated (12 Gy) or fractionated (3 Gy × 6 Gy) doses (103). When TAMs were collected from tumor-bearing mice after RT and transferred to other tumor-bearing mice, tumor growth was accelerated to a higher extent than with unirradiated-macrophage transfer (7). Altogether, these results showed that HDI skew TAMs in a M2-like phenotype (Table 1). This is why the blockade of M-CSF, in order to prevent macrophage recruitment, in combination with HDI is attractive. Indeed, it revealed interesting results: M-CSF inhibition combined to RT promotes tumor regression. This reinforces the idea that HDI-irradiated TAMs take part to the tumor growth and tumor radioresistance. Furthermore, high levels of TAM-derived IL-10 elicited T cell anergy in irradiated tumor (103). This is why an adoptive CD4^+^ T cell transfer has not met any success, except with the use of anti-M-CSF therapy combined to RT. This observation highlights the necessity to reprogram macrophages toward a M1-like phenotype to overcome the immunosuppression and the tumor regrowth after RT.

**Table 1:** Macrophage reprogramming after HDI, MDI, and LDI.

	Reference	Dose	Radiation type	Effect on polarization	Model
HDI	(7)	4 Gy × 5 × 3 week = 60 Gy	X-ray	M2 polarization	Mouse TRAMP-C1 (prostate)
	(101)	20 Gy × 3/day	X-ray	M2 polarization	Mouse Panc02 (pancreas)
	(102)	12 Gy	X-ray	M2 polarization	Human oral cancer (OSC-19 cells)—xenograft
	(103)	12 Gy or 3 × 6 Gy	X-ray	M2 polarization	Orthotopic pancreatic ductal adenocarcinoma model pancreas

MDI	(108)	2 Gy × 5 (10 Gy)	X-ray	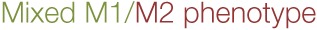	Human monocyte-derived macrophages (hMDM) with RKO or SW1463 cells
	(105)	5–10 Gy	X-ray	Increased inducible nitric oxide synthase (iNOS)/nitric oxide (NO) production	Raw 264.7 stimulated with lipopolysaccharide (LPS)/interferon γ (IFNγ)
	(104)	2 Gy × 5	X-ray	Reduced M2 markers	hMDMs
	(107)	2–4 Gy	γ-ray	M1 polarization	Raw 264.7, THP-1, hMDM
	(33)	2 Gy	γ-ray	M1 polarization	Raw 264.7 and peritoneal macrophages from RT5 mice
	(96)	2 Gy	γ-ray	M1 polarization	Tumor-associated macrophages from RT5 mice + CD8 T cell transfer (pancreas)
	(118)	1–2 Gy	X-ray	M2 polarization	Peritoneal macrophages from BALB/c mice + LPS/Raw 264.7

LDI	(105)	0.3–0.6–1.25 Gy	X-ray	Decreased iNOS/NO production	Raw 264.7 stimulated with LPS/IFNγ
	(109)	0.5–1 Gy	X-ray	M2 polarization	Raw 264.7
	(110)	0.5–0.7 Gy	X-ray	M2 polarization	THP-1 monocytes + LPS and monosodium urate crystals
	(111)	0.5–0.7 Gy	X-ray	Reduced M1 markers	THP-1 monocytes + LPS and monosodium urate crystals
	(112)	0.01–0.7 Gy	X-ray	M2 polarization	Peritoneal macrophages/Raw 264.7

*Red shading/font means M2 polarization, green shading/font means M1 polarization. HDI, high doses of irradiation; MDI, moderate doses of irradiation; LDI, low doses of irradiation*.

#### Moderate Doses of Irradiation (MDI)

*In vitro*, human unpolarized monocyte-derived macrophages shifted toward M1-like macrophages after MDI (2 Gy × 5). This is highlighted by the upregulation of pro-inflammatory markers (M1 phenotype) such as human leukocyte antigen-cell surface receptor (HLA-DR) and CD86, but also by the downregulation of anti-inflammatory markers (M2 phenotype) such as decreased mRNA expression of CD163, MRC1 (C-type mannose receptor 1, CD206) and versican (ECM proteoglycan) and reduced secretion of IL-10. In these macrophages, phagocytosis, associated with the M1-like phenotype, was increased after MDI while irradiation did not influence the ability of cocultured macrophages to promote the invasion of cancer cells and angiogenesis, features of M2-like macrophages (104). In the same line, unpolarized Raw 264.7 macrophages exhibited a higher expression of M1 markers (iNOS, TNFα, IL-12p70, IFNγ RANTES, and MCP-1) and a higher abundance of the phosphorylated p65 subunit from NFκB after γ-irradiation (2 Gy) (105). The γ-irradiation (2 Gy) of a human macrophage cell line (U937) also provoked a higher expression of TNFα and IFNγ compared to unirradiated macrophages (106). In another study, CD11b^+^/Gr-1 peritoneal macrophages from RT5 mice were γ-irradiated (2 Gy) *ex vivo* and showed an increased iNOS expression, related to M1 phenotype (33). Finally, PMA-differentiated macrophages (THP-1), murine macrophages (Raw 264.7) and human monocyte-derived macrophages (hMDM) revealed a pro-inflammatory profile after moderate doses of γ-rays (2 and 4 Gy). This was evidenced by increased mRNA levels for TNFα, IFNγ, IL-6, and IL-23 and higher protein levels for IL-1β and IL-8. While most of the previous studies showed the activation of NFκB p65 for macrophage reprogramming, this last study revealed that IR-induced M1-like phenotype was promoted by the transcriptional expression of IRF5 and was ataxia telangiectasia mutated (ATM)-dependent (107). Altogether, the results from *in vitro* experiment showed that unpolarized macrophages tend to acquire a M1 phenotype after MDI.

In addition to program unpolarized macrophages to M1 phenotype, MDI also potentiated the already acquired M1 phenotype. Indeed, MDI (2 Gy × 5) promoted a pro-inflammatory profile in M1 macrophages stimulated with LPS/IFNγ, as indicated by an increased expression of HLA-DR (104). Another *in vitro* experiment revealed that doses under 1 Gy prevented the polarization of macrophages toward M1 phenotype whereas doses of 5 and 10 Gy promoted the M1 phenotype in murine Raw 264.7 macrophages when stimulated with LPS/IFNγ. This is emphasized by an increased expression of iNOS and a higher NO production in irradiated macrophages (105). In contrast, in hMDMs stimulated with M-CSF and IL-10 (M2 macrophages), MDI did not influence the expression of pro- and anti-inflammatory markers (104). In other words, the effect of MDI on polarized macrophages showed an enhanced M1 phenotype in M1 macrophages but no change in M2 macrophages, meaning that MDI could not reprogram TAMs *in vitro*.

Cocultured experiments were performed between radiosensitive (RKO cells) or radioresistant (SW1463 cells) colorectal cancer cell lines and human unpolarized monocyte-derived macrophages. After irradiation, the mRNA expression of some pro-inflammatory (CCR7, IL1β, CXCL8) markers was decreased whereas the mRNA expression of anti-inflammatory markers was unchanged when macrophages were cocultured with RKO cells. MDI also promoted cancer cell invasion and migration of cocultured RKO cells. However, when macrophages were cocultured with SW1463 cells, there was an upregulation of the expression of pro-inflammatory (CCR7, CD80) and anti-inflammatory markers (IL-10 and CCL18) but no change of cancer cell migration and invasion. It means that following MDI, unpolarized macrophages adopt a different phenotype according to the type of cancer cells with which they interact (108).

Interestingly, *in vivo* experiments revealed promising results. A group of researchers suggested that moderated single dose (2 Gy) of γ-ray was able to completely reprogram TAMs in tumor. Klug and Prakash analyzed M1 (iNOS protein level, NO production) and M2 (Ym-1, Fizz-1 and Arg 1 protein level) markers in CD11b^+^ peritoneal macrophages from RT5 mice after whole-body irradiation (WBI). While untreated mice exhibited elevated expression of M2 markers in peritoneal macrophages, whole-body irradiated mice revealed increased M1 marker expression and decreased M2 marker expression. However, local irradiation was not able to modify the expression of M1 and M2 markers when measured in tumor tissue lysate. As local MDI (1, 2, and 6 Gy) induced a decline of T cell infiltration, the authors used CD8^+^ T cell transfer in addition to local irradiation (2 Gy) to induce a shift in the polarization of TAMs toward a M1 phenotype and to promote T cell infiltration in tumor. Local irradiation in combination with CD8^+^ T cell transfer induced an enhanced expression of IL-12p40 and IFNγ (M1 markers) and a reduced expression of IL-10 (M2 marker) in tumor tissue lysate. Indeed, the infiltration of tumor by T cells was exclusively dependent on the reprogrammed TAMs. When TAMs was depleted with CL treatment before local irradiation and CD8^+^ T cell transfer, there was an inhibition of T cell recruitment into irradiated tumors. The infiltration of CD8^+^ T cell into tumor is made feasible with the normalization of the vasculature. Indeed, pro-inflammatory macrophages elicited the normalization of the vasculature and allowed an efficient T cell transfer, leading to the eradication of the tumor and an increased survival in irradiated RT5 mice. However, it remains elusive how CD8^+^ T cell transfer impacts TAM reprogramming. Interestingly, local irradiation alone is not able to reprogram TAMs toward a M1-like phenotype. Then, it seems clear that CD8^+^ T cell transfer plays a role in macrophage reprogramming. The underlying mechanism needs to be further investigated (96) Table 1 summarized the effects of MDI on macrophage polarization described here above.

#### Low Doses of Irradiation (LDI)

Low doses of irradiation represents a good alternative in order to bypass the toxicities observed during intensive radiotherapy (HDI) (Table 1). However, LDI rather favor the M2 phenotype of TAMs. Indeed, LDI decreased the iNOS level and the NO production in Raw 264.7 macrophages polarized in M1 phenotype, resulting in repolarization of M1 macrophages toward M2 phenotype (105). Other results in favor of a M2-like phenotype were described after low doses of γ-radiation in murine macrophages. Indeed, irradiation of murine macrophages (RAW 264.7) with a dose of 0.5 Gy inhibited MPK1 [mitogen-activated protein kinase (MAPK) phosphatase]. MPK1 dephosphorylates the MAPKs p38, c-Jun and ERK1/2 and the dephosphorylation of p38 was associated with a decrease in TNFα and IL1β levels. Irradiation (0.5 Gy) of RAW264.7 macrophages stimulated with LPS also led to a decrease in p38 phosphorylation and TNFα production (109). In addition, Frey and Lodermann showed that low doses of X-rays (0.5–0.7 Gy) induced an anti-inflammatory phenotype in LPS-activated human THP-1 cells as evidenced by reduced amounts of secreted IL-1β in the medium. This was linked to a lower p65 NFκB nuclear translocation and a weak p38 phosphorylation (110, 111). Closely related to these results, it was also demonstrated that doses under 2 Gy favored the repolarization of M1 macrophages into M2-like macrophages, as evidenced by an increased level of TGFβ in the culture medium. This was associated to a reduced nuclear translocation of p65 NFκB (112).

In conclusion, HDI and LDI show no effect on M2 to M1 macrophage polarization while MDI clearly evoke TAM reprogramming. However, most of studies performed in this field are *in vitro* experiments, i.e., macrophages alone with no contact with cancer cells. Further *in vivo* experiments should reinforce the results obtained by Klug et al. with MDI. Furthermore, to our knowledge, no clinical study has been designed to analyze the effect of MDI on TAM reprogramming. It is thus difficult to transpose these data to the human tumors.

#### Whole-Body Irradiation

*In vivo* studies showed that exposure to low doses of WBI in healthy mice activated the innate immune cells including macrophages (113–115) (Table 2). In healthy mice, a single dose of WBI resulted in a T_H_1 cytokine expression profile whereas fractionated dose irradiation drove a T_H_2 shift in spleen and blood of mice (116). In the same line, single low doses (0.075 and 2 Gy) of total body radiation in healthy mice polarized peritoneal macrophages toward M1 phenotype as indicated by increased IL-12 and IL-18 secretion. This polarization is strongly linked to the activation of p65 NFκB and MyD88 a few hours after WBI (117). Only a few studies were published on WBI of tumor-bearing mice, as this treatment is irrelevant for human. However, as WBI would reprogram TAMs, studying the underlying mechanisms could be very helpful. A first study indicated that low doses (0.01–0.1 Gy) of WBI induced opposite effects on macrophages and NK cells in BALB/c radiosensitive mice and C57BL/6 radioresistant mice. Peritoneal macrophages exhibited a M1 phenotype in BALB/c mice, evidenced by an increased production of IL1β, IL-12 and TNFα. However, M2 macrophages were observed in tumor from C57BL/6 mice after WBI, partly elucidating the radioresistance of this mouse strain (118). Klug et al. also showed that peritoneal macrophages from RT5 mice, irradiated with a systemic dose of 2 Gy, revealed a higher iNOS level and NO production whereas the expression of M2 markers was decreased in these macrophages, indicating macrophage reprogramming. Furthermore, macrophages from total body irradiated mice, when transferred to tumor-bearing mice in parallel with CD8^+^ T cell adoptive transfer, induced tumor regression, similar to local irradiation in combination with CD8^+^ T cell adoptive transfer (96). In parallel, Prakash et al. described a switch of TAM polarization toward M1 phenotype when RT5 insulinoma bearing mice were treated by systemic irradiation (2 Gy/week on 2 weeks). Indeed, tumor lysates presented a higher expression of M1 markers [iNOS, pSTAT3, TNFα, IL-12 (p70)] and a lower expression of M2 markers (CD206, Fizz-1, Ym-1 and Arg1). Peritumoral macrophages showed an elevated iNOS expression and NO production after WBI, modifying the tumor microenvironment with the normalization of the tumor vasculature.

**Table 2:** Macrophage reprogramming after WBI.

Reference	Dose	Radiation type	Effect	Model
(113)	0.2 and 2 Gy	γ-ray	M1 polarization	WBI (without tumor)
(117)	0.075 and 2 Gy	X-ray	M1 polarization	WBI (without tumor)
(114)	0.5–6 Gy	X-ray	M1 polarization	WBI (without tumor)
(118)	0.01–0.02–0.1 Gy × 10/day × 2 weeks (LDI)	X-ray	Increased cytotoxic activity and nitric oxide	Macrophages from BALB (L1)/c and C57BL/6 (Lewis lung cancer) mice (WBI)
(96)	0.5–2 Gy × 1 (MDI)	X-ray	M1 polarization	Mouse RT5 insulinoma/Human MeWo melanoma xenograft/Human pancreas
(33)	2 Gy × 2 (MDI)	γ-ray	M1 polarization	Rip1 Tag5 tumor-bearing mouse (WBI)

*Green shading means M1 polarization. WBI, whole-body irradiation*.

To explain the discordance between local irradiation and WBI on the reprogramming of TAMs, the authors suggested that WBI allowed the mobilization of fresh reprogrammed macrophages from various lymphoid organs to infiltrate the tumor site and the surrounding microenvironment. Infiltration of tumor by reprogrammed macrophages can result in the activation of antitumor T-cell responses and can thereby act as an “endogenous vaccine” (33) On the other hand, local irradiation is not able to invite macrophages from lymphoid organs to the tumor site. It could be hypothesized that the transfer of CD8^+^ T cells would induce macrophage reprogramming in lymphoid organs. The combination of T cell transfer to irradiation would promote the normalization of the tumor vasculature, allowing tumor perfusion by CD8^+^ T cells and reprogrammed macrophages.

### Molecular Pathways Responsible for the Repolarization of TAMs after RT

There are several mechanisms proposed to explain the influence of the irradiation dose on the polarization of TAMs. These mechanisms are partly different from those involved in the reprogramming mediated by chemotherapy and immunotherapy, and they include ROS, DNA damage, p50–p65 NFκB activation, and MAPK phosphorylation. The differences between the different doses could be due to the activation of different pathways according to the dose, i.e., a switch in the NFκB subunit balance for moderate doses while high doses would induce apoptosis.

#### NFκB Balance

**Figure 3:**
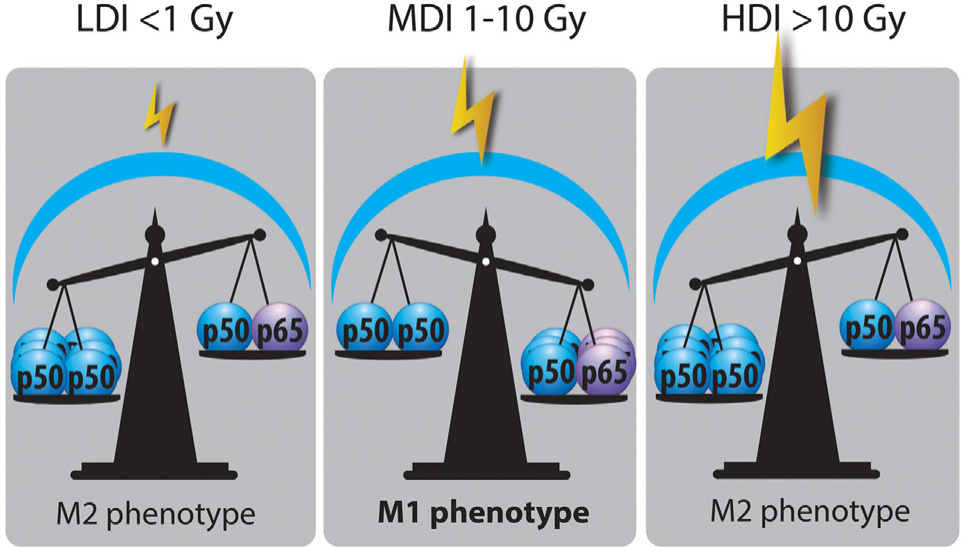
Nuclear factor kappa B (NFκB) balance state after LDI, MDI, and HDI. Irradiation dose showed opposite effects on NFκB balance in macrophages: LDI or doses lower than 1 Gy did not modify the abundance of p50–p50 NFκB in macrophage nucleus after radiation. MDI or doses from 1 to 10 Gy induced a switch of NFκB balance from the inactive homodimer (p50–p50) to the active heterodimer (p50–p65), correlated with a reprogramming of tumor-associated macrophages (TAMs). HDI or doses higher than 10 Gy were not able to change the NFκB balance, skewing TAMs in a M2 phenotype (HDI, high doses of irradiation; MDI, moderated doses of irradiation; LDI, low doses of irradiation).

Nuclear factor kappa B plays an important role in the polarization of macrophages. It was shown that M2 macrophages acquired their phenotype under the effect of p50–p50 NFκB homodimer while M1 macrophages are answerable to p50–p65 NFκB heterodimer (24). Once p65–p50 NFκB is translocated into the nucleus, it allows the expression of pro-inflammatory mediators [TNFα, IL-1β, IL-6, IL-12(p40), IFNγ, CXCL10 and NOS2 (14)] whereas p50–p50 NFκB inhibits the expression of these genes. Indeed, several experiments revealed the need for p65 translocation to activate M1 polarization (24, 119). Crittenden and his colleagues evidenced that HDI (60 Gy) produced M2 phenotype through p50–p50 dimer activation, leading to a subsequent IL-10 production (101). In the same line, low X-ray radiation dose (<1 Gy) reduced the translocation of p50–p65 NFκB in M1 activated macrophages (112). LDI on human macrophages and LPS-activated THP-1 macrophages reduced the nuclear amount of p65 NFκB, a phenomenon also correlated to a decreased secretion of IL-1β (M1 cytokine) (110). Interestingly, Teresa Pinto et al. revealed an enhanced phosphorylation of RelA (p65) in macrophages following MDI (2, 6, and 10 Gy), correlated to M1 repolarization (104). Hildebrandt et al. also evidenced the role of NFκB in TAM reprogramming in Raw264.7 macrophages after 2 Gy irradiation (105). In an *in vivo* study, whole-body γ-irradiation (2 Gy)-induced the phosphorylation of NFκB p65 in tumor lysate. The activation of NFκB p65 is closely related to a reprogramming of TAMs toward M1-like macrophages (33) (Figure 3).

In sepsis, a sustained activation of macrophages by LPS leads to a tolerance and a reprogramming of macrophages toward M2 phenotype. This reprogramming is driven by p21, involved in the shifting of the balance between p65–p50 and p50–p50 NFκB dimers. Indeed, p21 deficiency is linked to a reduced DNA-binding affinity of the p50–p50 homodimer and a prevalence for the p65–p50 heterodimer in macrophages stimulated with LPS (120, 121). In some cases, ubiquitination is controlled by the phosphorylation of a protein substrate (122). Indeed, some effectors of the DNA repair machinery are described to be involved in the ubiquitin proteasome pathway and regulate p21 protein level in ML-1 cells (myelocytic leukemia cell line) (123). Therefore, a high ubiquitination of p21 would lead to a lower level of p21 protein. This could be correlated with an increased p65–p50 DNA-binding affinity and could potentially favor a M1 phenotype after IR. Further investigations are, however, required to understand the possible role of p21 in regulating the NFκB balance after IR.

#### ATM Kinase

Radiotherapy induces major effects on cells by creating DNA damage. DNA damage and ROS produced by γ-radiation (2–20 Gy) induced the phosphorylation of ATM in U937 human macrophage cell line (106). When DNA damage appeared, ATM plays a central role in the detection and the activation of the DNA repair machinery [for review see Ref. (124)]. The activation of ATM notably leads to the ubiquitination of NEMO (NFκB Essential Modulator), a subunit of the IKK complex, a few hours after macrophage irradiation. The ubiquitination of NEMO is ATM-dependent as indicated by the reduced ubiquitination of NEMO when an ATM inhibitor (Ku5593) was used in combination with γ-rays (125). Ubiquitinylated NEMO then drives the activation of IKK complex in the cytoplasm. This activation goes through ubiquitination of IKβ and the subsequent degradation of this inhibitor by the proteasome. p65–p50 NFκB is, therefore, released in the cytoplasm and free to move into the nucleus where the heterodimer acts as a transcriptional factor [for review see Ref. (126)].

After γ-radiation (2 Gy), the phosphorylation of NEMO was followed by the activation of NFκB (p65). A subsequent upregulation of M1 markers (CD86, CD40, HLA-DR, TNFα, IFNγ) and a reduced secretion of IL-4 (M2 marker) followed the activation of NFκB. These changes are partially generated by elevated ROS content in cells, as indicated by the increase or decrease in the phosphorylation of NEMO when U937 monocytic cells were irradiated and treated with l-buthionine sulphoximine or *N*-acetyl cysteine (NAC), respectively. Indeed, l-buthionine sulphoximine irreversibly inhibits γ-glutamylcysteine synthase, hence depleting GSH level and increasing ROS content. NAC is a potent antioxidant, known to decrease ROS level by increasing GSH levels (106). However, another team showed that γ-rays (0, 3, 6, and 10 Gy) induced NFκB activation in Raw 264.7 macrophages, as highlighted by the degradation of the IKβ protein. The activation of NFκB was related to a higher NO production in macrophages. This phenomenon is dependent on DNA damage more than ROS level, since NAC did not affect NO production and IKβ degradation in irradiated cells (127).

Moreover, modulators of DNA repair, such as Olaparib (PARP inhibitor), increased ATM activation and upregulated IRF5 transcription, resulting in macrophage activation toward a pro-inflammatory phenotype (107). In conclusion, IR is able to induce DNA damage by direct or ROS-dependent interactions with DNA. DNA damage leads to the recruitment of the ATM kinase and DNA repair machinery. Consequently, ATM contributes to the reprogramming of macrophages after irradiation.

#### Reactive Oxygen Species

Radiation therapy promotes the formation of radicals, such as ROS or RNS in cells. ROS quantities depend on the dose applied to the cells. In macrophages, different ROS concentrations can differently influence the cellular responses and the polarization of macrophages. Indeed, the role of ROS in the polarization of macrophages is not so clear but the most accepted idea is that ROS play key roles in M1 responses (e.g., defense against invading microbes) (128) and regulates M2 polarization (129, 130).

##### M1 Polarization

The polarization of macrophages toward M1 or M2 phenotype goes through several pathways, including NFκB. There is a balance between the inhibitory heterodimer NFκB (p50–p50) and the active heterodimer NFκB (p50–p65) (24). When this balance leans on one side or the other, macrophages undergo M2 or M1 polarization, respectively. It is also well known that ROS generation mediated the production of pro-inflammatory cytokines (131). Indeed, this ROS production activates MAPK and NFκB pathways (132). It was shown that H_2_O_2_ enhanced p65 NFκB DNA-binding and promoted p65 NFκB phosphorylation. This NFκB activation is followed by its translocation into the nucleus in macrophage and monocytic cell lines, and then induces M1 polarization (133). In parallel, it was shown that H_2_O_2_ altered protein–protein interaction of p50–p50 NFκB, therefore reducing p50–p50 NFκB activation, supporting M1 activation (134). As previously said, ROS production is observed after irradiation. This is due to the direct ionization of molecules by incident photons. Another way for γ-irradiation to induce ROS generation is the activation of NADPH oxidase (NOX). The NADPH oxidase (NOX 1 and NOX 2) catalyzes the oxidation of nicotinamide adenine dinucleotide in the NADP^+^ + H^+^ while reducing O_2_ in ${\text{O}}_{2}^{-}$, leading to the formation of H_2_O_2_. γ-irradiation (2 Gy) promoted ROS production in Raw264.7. This ROS production was partly inhibited by the use of NAC and by diphenylene iodonium, a NOX inhibitor. In parallel, the protein expression of NOX2 was increased after γ-irradiation in PMA-differentiated THP-1 cell and in Raw264.7 macrophages. Altogether, these results indicated that moderate doses of γ-irradiation generated NOX2-dependent ROS production. Elevated ROS content yielded ATM activation, as suggested by the absence of ATM phosphorylation in presence of NAC or siNOX2 when Raw264.7 macrophages were irradiated. This NOX2-dependent ATM activation controlled the polarization of Raw 264.7, THP-1 and hMDM in M1-like macrophages, through the regulation of IRF5 transcription. Moreover, in patients, the perturbation of ATM-dependent NOX2 signaling pathway was associated with a decreased iNOS macrophage number and poor tumor responses to radiotherapy (107).

##### M2 Polarization

Not only, NAPDH oxidase activity drives M1 polarization, but its inhibition conversely triggers M2 polarization. A previous study showed that the inhibition of NOX promoted anti-inflammatory microglial activation (M2 phenotype) during neuroinflammation (135). Furthermore, another group revealed that the deletion of NOX1/2 reduced ROS production during macrophage differentiation. This double knockout is not critical for M1-like polarization of mouse bone marrow-derived macrophages, but prevented M2 polarization, as evidenced by the reduced expression of mCCL17, mCCL24 and RELMα. Indeed, NOX1/2 is crucial for the activation of c-Jun N-terminal kinase (JNK) and extracellular signal-regulated kinase (ERK) MAPKs during monocyte to macrophage differentiation and then affects M2 polarization (136). High concentrations of ROS play a decisive role in the differentiation and the polarization of macrophages into M2 phenotype and antioxidants treatment affected M2 but not M1 macrophage polarization. The use of butylated hydroanysole and other ROS scavengers repressed tumorigenesis by blocking the occurrence of TAMs (129). H_2_O_2_ stimulates the production of TNFα and activates the transcription factor STAT6, responsible for the expression of M2 markers such as Fizz-1 in macrophages (137, 138).

All these data showed that high concentrations of ROS trigger M2 polarization while smaller concentrations are responsible for M1 polarization. On the one hand, the production of ROS driven by HDI prevents the reprogramming of TAMs toward M1-like macrophages. On the other hand, LDI induce small ROS production, probably not enough to reach the range of concentrations able to reprogram macrophages. However, MDI allows to reach the right range of ROS concentrations in macrophages to activate p65 NFκB, IRF5, and MAPK notably through ATM activation, hence favoring M1 phenotype.

Further investigations are needed to better understand the effect of IR on the ROS generation in TAMs and the molecular pathways involved in the polarization of macrophages *via* ROS production.

#### IRF5

Interferon-regulatory factor/signal transducer and activator of transcription (IRF/STAT) signaling is a central pathway in macrophage polarization. IRF can interact with two adaptors, MyD88 and TRIF, downstream of cytokine receptors or TLR. IRF5 and IRF4 are in competition to the binding of MyD88 and the subsequent activation of pro-inflammatory transcription factors, including NFκB. The activation of IRF5 through MyD88 then promotes M1 phenotype in macrophages. As a competitor for MyD88, IRF4 acts as an antagonist and can suppress M1 macrophage polarization. The balance between IFR4 and IRF5 in cells is critical for M2 and M1 polarization, respectively (24, 139).

Only one *in vitro* study established the role of IRF5 in macrophage reprogramming after irradiation. In this study, the authors showed that macrophages acquired a M1-like phenotype after exposition to γ-irradiation (2 Gy). This polarization is made feasible by the activation of NOX2 and ATM, hence driving the transcription of IRF5 (107). While these results are very interesting, the contribution of IRF/STAT pathway to macrophage polarization mediated by irradiation needs to be further investigated *in vivo* and in patients. Furthermore, IRF5 modulation is also known to drive M1 polarization through the activation of NFκB. The link between IRF and NFκB in macrophage reprogramming after radiotherapy should also be considered.

#### iNOS Level and NO Production

Nitric oxide mediator is a double-edge sword key regulator in tumor progression. On the one hand, NO has antitumoral effects as it promotes DNA damage, elicits cell death, and enhances anticancer therapeutic efficacy. Indeed, patients with lower iNOS expression levels, hence low NO production, showed recurrence of tumors and metastasis after RT. On the other hand, NO mediator is also known for its pro-tumoral effects since it induces antiapoptotic effects and promotes cell cycle, cancer progression, metastasis, angiogenesis, and chemoprotection. High level of iNOS is associated with poor prognosis and tumor-promoting effects (140). It thus seems that critical NO concentrations and iNOS levels finely modulate the fate of the tumor.

High doses of irradiation induced NO production and high iNOS expression level in macrophages. For instance, high doses of γ-irradiation (20 Gy) led to iNOS activation and NO production, favoring tumor growth and recurrence after RT (141). Another *in vivo* study using high doses (25 Gy or 3 Gy × 20 Gy) also generated high level of iNOS in murine macrophages after IR, contributing to the tumor growth (7). NO production contributes to DNA repair by inducing p53, poly(ADP ribose) polymerase and DNA-dependent protein kinase activation, leading to tissue regeneration and tumor regrowth. In addition, NO mediator is related to tumor angiogenesis and promotes reoxygenation. Therefore, targeting iNOS in murine macrophages enhanced postradiotherapeutic efficacy after HDI (141).

On the other hand, WBI (2 Gy × 2 Gy) on RT5 mice induced an increased NO production and iNOS level, enough to induce tumor regression. In addition, *ex vivo* radiation with similar doses on peritoneal macrophages from RT5 mice also stimulated an increased in iNOS level and NO production (33). Indeed, NO can be considered as a free radical and as a source of reactive oxygen and nitrogen species, inducing DNA damage (142). Another team hypothesized that iNOS and NO production induced by MDI (2 Gy) remodeled vasculature allowing cytotoxic lymphocyte recruitment, and subsequent tumor eradication (15). NO leads to higher tumor oxygenation by promoting vasodilatation and hence radiosensitization (142). It can diffuse toward bystander cancer cells leading to their radiosensitization (143).

High doses of irradiation would induce high level of NO in macrophages, leading to cancer progression. Contrarily, MDI drive a slight increase in NO production in macrophages, enough to participate to the reprogramming of TAMs and favoring tumor regression.

#### MAPK (p38–ERK–JNK)

Mitogen-activated protein kinases are serine threonine kinases activated by diverse stimuli including cytokines and ROS. MAPKs regulate diverse pathways such as cell proliferation, apoptosis, and differentiation. MAPKs include extracellular signal-regulated kinase 1 and 2 (ERK 1/2), JNK and p38. They mediate immune cell functional responses to diverse signals and play a role in macrophage polarization. MAPK kinases (MAPKKs) activate MAPKs through dual phosphorylation on tyrosine and threonine residues (Thr–X–Tyr motif) located in the activation loop of the kinase domain (144). The phosphorylation of MAPK activates pro-inflammatory gene transcription and is correlated with M1-like macrophages. At the opposite, MAPKs are inactivated through dephosphorylation of threonine and/or tyrosine residues within the activation loop by MAPK phosphatase (MKP-1) (145). MKP-1-deficient macrophages exhibit skewed activation profiles: macrophages showed an enhanced pro-inflammatory phenotype in response to IFNγ and TNFα while this deficiency severely suppressed “M2-like” phenotype after IL-4 stimulation (146). Compared to wild-type mice, MKP-1-deficient mice produced greater amounts of TNFα, IL-1β, CCL2, granulocyte/macrophage colony-stimulating factor (GM-CSF), IL-6, IL-10, and IL-12p70 (147, 148).

Mitogen-activated protein kinases are also regulated by ROS and DNA damage (132). Within a few minutes, low doses (0.5 and 1 Gy) of γ-radiation induced a dephosphorylation of both ERK 1/2 and p38 MAPKs. This dephosphorylation depended on MKP-1 and induced the suppression of TNFα production in Raw 264.7 cells. These results correlated with the inability of LDI to reprogram M2 macrophages (109). Another study showed enhanced phosphorylation of ERK 1/2 and p38 following moderated doses (2 Gy) of γ-radiation in resident peritoneal exudate cells. This activation was related to a decreased dephosphorylation exerted by MKP-1 and was correlated with TNFα production. The explanation relies on the oxidation of the catalytic cysteine of MKP that inactivates the phosphatase, then triggering the activation of MAPK cascade including JNK and p38. These results correlated with the reprogramming of macrophages induced by MDI (149). Conflicting results revealed the inhibition of p38 phosphorylation in macrophages after WBI of tumor-bearing mice. The inhibition of p38 phosphorylation in macrophages allowed the reprogramming of TAMs toward M1 phenotype in tumor. This inhibition could be linked to the upregulation of MKP-1 (33).

#### Nuclear Erythroid Derived 2-Related Factor (Nrf2)

The Nrf2 is involved in the management of intracellular oxidative stress. It is also known to repress inflammation by inhibiting pro-inflammatory cytokine expression. Indeed Nrf2 is able to bind the close proximity of IL-6, IL-1β, and IL-1α genes in LPS-stimulated macrophages. This binding hence inhibits the recruitment of RNA polymerase II to the transcription start site without affecting the p65 NFκB recruitment. The Nrf2 binding to these genes is independent on ROS level since the use of antioxidant (NAC) did not affect the inhibition of pro-inflammatory gene transcription mediated by Nrf2 (150). The transcription factor Nrf2 was translocated into the nucleus even after 0.1 Gy γ-radiation, leading to the upregulation of antioxidant proteins such as heme oxygenase-1 in Raw 264.7. Nrf2 translocation increased with the dose (0.1–2.5 Gy). The regulation of Nrf2 is mediated by ERK 1/2 pathway after the irradiation of macrophages (121). It is also known that in oxidative stress conditions, p21 interacts with Nrh2 domain of Nrf2, upregulating Nrf2-mediated antioxidant response (151). This could link the Nrf2 pathway to NFκB in the reprogramming of TAMs after IR: the absence of p21 prevents the anti-inflammatory response mediated by Nrf2 and favors the p50–p65 NFκB DNA-binding. The fate of irradiated macrophages would then depend on the connection between ROS level/DNA damage and the downstream-activated pathways.

### Overall View Regarding TAM Reprogramming upon Radiotherapy

Local radiotherapy can finely tune the balance between immunosuppression and immune antitumor properties. The shift toward one or the other immune state depends on the dose per fraction, the total dose and the cancer type. Whereas local IR finds difficulties to completely reprogram TAMs, WBI can be helpful in the understanding of TAMs reprogramming since it activates the immune system outside the tumor site, leading to the complete eradication of the tumor. It, thus, seems that local IR needs something more than its sole effects on macrophages to induce a total reprogramming of TAMs and to restore a full immune antitumor response.

**Figure 4:**
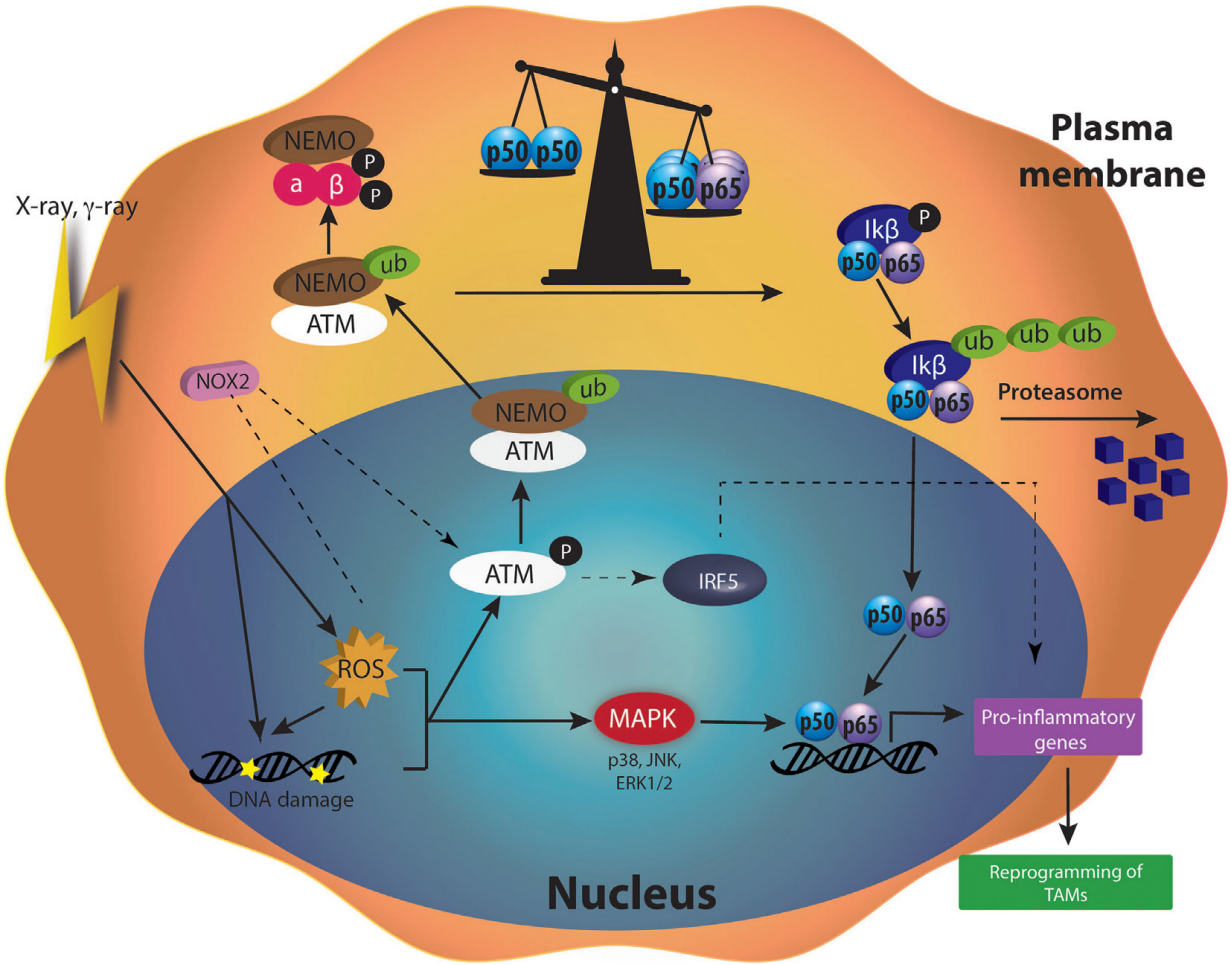
Molecular mechanisms activated after moderated doses of irradiation in tumor-associated macrophages (TAMs). Ionizing radiation (X-rays or γ-rays) induces DNA damage and elevated reactive oxygen species (ROS) content in cells. DNA repair machinery [such as ataxia telangiectasia mutated (ATM)] is activated by DNA damage and initiates the ubiquitination of NFκB essential modulator (NEMO), a subunit of the IKK complex. Therefore, ubiquinated NEMO can drive the activation of IKK complex in the cytoplasm. The degradation of IκB protein by the proteasome allows the release of p50–p65 nuclear factor kappa B (NFκB) in the cytoplasm. p50–p65 NFκB is then translocated into the nucleus and induces the transcription of pro-inflammatory genes, leading to the reprogramming of TAMs. ROS are also able to stimulate mitogen-activated protein kinase (MAPKs). Once phosphorylated, MAPKs also participate to the activation of NFκB and hence, to the transcription of pro-inflammatory genes.

The mechanisms underlying macrophage reprogramming after radiation therapy are summarized in Figure 4. This figure emphasizes the central role of NFκB in macrophage reprogramming after MDI. Although most of these studies described in this review are promising, there is still a huge need for further investigations and *in vivo* confirmations. Indeed, cancer cell irradiation generates damage-associated molecule patterns (DAMPs), such as high mobility group box 1 (HMGB1). DAMPs bind on their corresponding pattern-recognition receptor, such as TLR4 for HMGB1 in macrophages, triggering a pro-inflammatory phenotype (152). The irradiation then promotes antitumoral responses. However, it is possible that the contact between macrophages and cancer cells could offer the response of macrophages to radiotherapy. Indeed, cancer cells secrete different cytokines and chemokines that could influence the faith of macrophage polarization. More precisely, the type of cancer cells in the tumor can modulate macrophage reprogramming after irradiation, as evidenced by the opposite results acquired with different cancer cell lines in cocultured with macrophages (108).

In parallel, IR can also alter other types of cells in tumor, such as lymphocytes and dendritic cells, as well as the tumor vascularization (152). For example, ablative radiation therapy promotes CD8^+^ T cell accumulation into the tumor (153, 154). Indeed, single moderate doses (10 Gy) of irradiation mobilized CD8^+^ T cell through dendritic cells activation hence reducing or eradicating tumor. Therefore, MDI fulfills mobilization of effectors from the immune system and is able to reprogram TAMs in an antitumor phenotype. However, the issue about the fractionation should also be addressed, as in patients the treatments are usually given in several doses. It was shown that hyperfractionation (5 Gy × 4 Gy) could completely abrogate the effect on CD8^+^ T cell mobilization observed with single high dose (20 Gy) (154). The effects of fractionation on macrophage reprogramming remain elusive and need to be further investigated.

## Conclusion and Future Directions

Chemo- and immunotherapies, as well as moderate dose of irradiation are able to reprogram TAMs into M1-like macrophages. TAM reprogramming is induced through various mechanisms. These pathways mainly include NFκB, NO production, and STAT1 that can act together. MDI-induced reprogramming is made possible through DNA damage-dependent ATM activation, the production of a specific range of ROS and NO amounts, a shift of the NFκB balance to the active p50–p65 heterodimer, the transcription of IRF5 and the activation of the MAPK pathway. Amongst them, NFκB pathway seems to be a central target for macrophage reprogramming.

Local IR is able to kill cancer cells and to activate the immune system by the release of cancer cell antigens and of immune-associated factors by stromal cells and vascular endothelial cells, but it also depletes T cells and antigen-presenting cells (155). Combined radiotherapy and immunotherapy have shown promising results in reprogramming macrophages and may thus be useful for tumor elimination. However, the choice of T cell adoptive transfer can determine the fate of the tumor and macrophage reprogramming, as CD8^+^ T cell adoptive transfer is more effective than CD4^+^ T cell adoptive transfer (96, 103). Indeed, the use of a anti CD4^+^ T cell therapy combined to γ-irradiation (5 Gy) in a mammary mouse model improved the efficacy of radiotherapy (156). The use of MDI combined to adoptive CD8^+^ T cell transfer or stimulating CD8^+^ T cell therapy (such as anti PD-L1/programmed cell death 1) may give promising results as effective anticancer therapies. The transfer of irradiated macrophages was also the subject of numerous studies in murine models. However, human clinical studies using transferred macrophages have also been performed but showed no or few improvements regarding cancer regression (157).

Another avenue to explore would be the effect of high LET particles, for example protons, since protontherapy is more and more developed in clinic. As the mechanism of action is different from that of conventional radiotherapy (X-rays), the effects on macrophage reprogramming could be potentiated. Indeed, charged particles can drive huge NFκB activation compared to X-rays or γ-rays, as shown in HEK/293 cells (158).

Targeting the immune system in cancer diseases demonstrated successful improvement on cancer elimination and has become a highly promising therapy. Therefore, MDI combined to chemo- or immunotherapies targeting macrophage reprogramming could also synergize their effects on tumor regression.

## Author Contributions

GG wrote the review and designed the figures and the tables. SL and CM supervised the whole work, contributed to writing, and critically revised the paper.

## Conflict of Interest Statement

The authors declare that the research was conducted in the absence of any commercial or financial relationships that could be construed as a potential conflict of interest. The reviewer, BB, and handling editor declared their shared affiliation, and the handling editor states that the process nevertheless met the standards of a fair and objective review.

## Abbreviations

AMPK, AMP-activated protein kinase; AP-1, activator protein 1; Arg 1, arginase 1; ATM, ataxia telangiectasia mutated; C/EBPβ, CCAAT-enhancer-binding proteins; CCL, chemokine ligand; CCR2, C–C chemokine receptor type 2; CD206, cluster of differentiation 206 or mannose receptor; CL, clodrolip; COX, cyclooxygenase; CpG-ODN, unmethylated cytosine-guanine (CpG) oligodeoxynucleotides; CSF-1, colony-stimulating factor 1; CSF-1R, colony-stimulating factor 1 receptor; CTLA4, cytotoxic T lymphocyte-associated protein 4; CXCL12, C–X–C motif chemokine 12; ECM, extracellular matrix; ERK, extracellular signal-regulated kinase, FDA, food and drug administration; FRβ, folate receptor β; HDI, high doses of irradiation; HIF, hypoxia-inducible factor; HLA-DR, human leukocyte antigen-cell surface receptor; HO1, heme oxygenase 1; HRG, histidine-rich glycoprotein; IFNγ, interferon γ; IL, interleukin; iNOS, inducible nitric oxide synthase; IR, ionizing radiation; IRF, interferon-regulatory factor; JNK, c-Jun N-terminal kinase; KLF4, Kruppel-like factor 4; LDI, low doses of irradiation; LET, linear energy transfer; LLC, Lewis lung cancer; LPS, lipopolysaccharide; mAb, monoclonal antibody; MAPK, mitogen-activated protein kinase; MARCO, pattern recognition scavenger receptor; MCP-1, monocyte chemoattractant protein 1; M-CSF, macrophage colony-stimulating factor; MDI, moderate doses of irradiation; MHC, major histocompatibility complex; MMP, matrix metalloproteinase; MnSOD, manganese superoxide dismutase; MPK1, MAPK phosphatase 1; MRC1, mannose receptor-C 1; NAC, *N*-acetyl cysteine; NADPH, nicotinamide adenine dinucleotide; NEMO, NFκB essential modulator; NFκB, nuclear factor kappa B; NK, natural killer; NO, nitric oxide; NOX, NADPH oxidase; Nrf2, nuclear erythroid derived 2-related factor; p38, MAPK; PAMPs, pathogen-associated molecular patterns; PD-1, programmed cell death 1; PD-L1, programmed death ligand 1; PDA, pancreatic ductal adenocarcinoma; PEC, peritoneal exudate cells; PI3Kγ, phosphoinositide 3-kinase gamma; PlGF, placental growth factor; PPARγ, peroxisome proliferator-activated receptor gamma; RANTES, regulated on activation, T cell expressed and secreted; RELMα, resistin-like molecule alpha; RNS, reactive nitrogen species; ROS, reactive oxygen species; RT, radiotherapy; STAT, signal transducer and activator of transcription; SUMO, small ubiquitin-like modifier; TAMs, tumor-associated macrophages; TGFβ, transforming growth factor β; TLR, toll-like receptor; TNFα, tumor necrosis factor α; TRAIL-R2, TNFα-related apoptosis-inducing ligand receptor; VCAN, versican, extracellular matrix proteoglycan; VEGF, vascular endothelial growth factor; ZA, zoledronic acid.
